# EEG Sensor-Based Parkinson’s Disease Detection Using a Multi-Domain Feature Fusion Network

**DOI:** 10.3390/s25237189

**Published:** 2025-11-25

**Authors:** Jinxuan Wang, Hua Huo, Shilu Kang, Lan Ma, Chen Zhang

**Affiliations:** College of Information Engineering, Henan University of Science and Technology, Luoyang 471023, China; wangjinxuan@stu.haust.edu.cn (J.W.); ksl_life@163.com (S.K.); 215400000023@stu.haust.edu.cn (L.M.); 250410040094@stu.haust.edu.cn (C.Z.)

**Keywords:** Parkinson’s disease detection, EEG sensor, deep learning, multi-domain fusion

## Abstract

Parkinson’s disease (PD) is a common neurodegenerative disorder, and accurate identification of PD is critical for clinical diagnosis and disease management. Electroencephalography (EEG) sensors provide reliable real-time brain signal acquisition, making them practical biosensing modalities for PD detection. However, due to their non-stationarity, single time-domain or frequency-domain analysis methods are insufficient to extract robust discriminative features from EEG signals. To address this challenge, we propose a multi-domain feature fusion EEG classification model, termed Multi-Domain Fusion Network (MDF-Net), which jointly integrates temporal, frequency-domain, and wavelet-domain representations for accurate PD recognition. MDF-Net employs a Temporal Attention-enhanced Temporal Convolutional Network (TTCN) to capture temporal dependencies and incorporates an improved 1D Convolutional Neural Network mixer module (Cmix) for multi-channel feature fusion. We constructed an EEG dataset of 415 subjects (289 healthy controls and 126 PD patients). Under 5-CV, the proposed method achieved a classification accuracy of 92.3%, an F1-score of 87.3%, and an AUC of 0.943. Experimental results demonstrate that multi-domain feature fusion effectively improves PD detection performance, and EEG sensor-based analysis shows strong potential for clinical application. This study provides a methodological reference for developing objective, practical computer-aided diagnostic tools for PD.

## 1. Introduction

Parkinson’s disease (PD) is the second most prevalent neurodegenerative disorder worldwide [1], and its incidence has shown a continuous upward trend in recent years [2,3]. Patients with PD commonly exhibit motor symptoms such as bradykinesia and gait disturbance, as well as non-motor symptoms including insomnia, depression, and olfactory dysfunction [4]. The typical neuropathological hallmark of PD is dopaminergic neuronal degeneration in the substantia nigra pars compacta [5]. Its underlying pathogenesis is believed to be associated with genetic factors, aging, environmental toxins, and brain injury [6,7], although a definitive mechanistic explanation remains unclear.

In recent years, increasing attention has been directed toward the relationship between PD and abnormal brain function [8], and substantial progress has been made in utilizing electroencephalography (EEG) to investigate PD. For example, Yi et al. [9] and Peláez et al. [10] explored the application of EEG in PD diagnosis and neuropathological analysis. In addition, some studies have employed EEG sensors for PD detection, as demonstrated by the work of Jibon et al. [11] and Siuly et al. [12]. However, EEG signals are inherently nonlinear, non-stationary, and prone to noise. These characteristics make signal analysis challenging. Even when analyzed in the time domain, effective feature extraction requires incorporating frequency-domain analysis and other advanced signal processing techniques [13].

To address these issues, we collected EEG data from 289 healthy controls (HCs) and 126 PD patients using a 32-channel EEG sensor system and proposed a multi-domain EEG analysis model named Multi-Domain Fusion Network (MDF-Net), which integrates temporal, frequency-domain [14], and wavelet-domain [15] features. The proposed model effectively extracts discriminative features from EEG signals across multiple domains. A Temporal Convolutional Network (TCN) [16], combined with a 1D-Convolutional Neural Network (CNN) channel fusion mechanism, is employed to capture cross-domain channel dependencies. This design ultimately enables accurate PD detection.

The main contributions of this study are as follows:

1. We construct a high-quality EEG dataset for PD analysis, consisting of 32-channel EEG recordings from 415 subjects (289 HC and 126 PD). Furthermore, we systematically analyze the effects of segment window size and sampling frequency on model performance, providing valuable data and methodological reference for EEG-based PD detection.
2. We propose a multi-domain information fusion deep learning model, MDF-Net, which combines temporal-, frequency-, and wavelet-domain feature extraction. MDF-Net utilizes a Temporal Attention-enhanced Temporal Convolutional Network (TTCN) to extract temporal dependencies, and a 1D-CNN mixer module (Cmix) to model multi-scale spectral and wavelet features, thereby effectively enhancing the representation of EEG non-stationarity and multi-frequency oscillations, which in turn improves PD identification robustness and generalization.
3. Extensive comparison and ablation experiments validate the effectiveness of the proposed MDF-Net and its three-domain feature fusion strategy. On the large-scale EEG dataset containing 415 subjects, MDF-Net achieves high detection accuracy and F1-score, demonstrating strong potential for practical application in PD detection.

The remainder of this paper is organized as follows. Section 2 reviews related work on EEG sensor-based PD detection. Section 3 describes the dataset, data preprocessing, and the proposed MDF-Net architecture. Section 4 presents the experimental settings and results. Section 5 discusses the performance evaluation, as well as the impact of segment window size and sampling frequency. Section 6 concludes the study and outlines future research directions.

## 2. Related Work

Research on sensor-based PD detection has primarily focused on various external and physiological signal acquisition methods, including inertial sensors [17], video sensors [18], electroencephalography (EEG) sensors [19], and plantar pressure sensors [20]. Inertial sensors capture human kinematic parameters and are often used to characterize motor dysfunction in PD patients. For instance, Sánchez-Fernández et al. [21] developed a fuzzy inference model based on inertial sensor data for PD recognition, whereas Son et al. [22] distinguished PD patients from HC by analyzing differences in motion signal patterns. In comparison with wearable inertial sensors, video sensors enable contactless motion assessment. Ma et al. [23] extracted gait features from video signals for PD detection, while Acevedo et al. [24] focused on hand motion video analysis to identify PD-related impairments.

However, in contrast to externally measured movement-related data, EEG signals provide greater research value because the core pathological mechanism of PD originates from neurodegenerative changes in the central nervous system, and EEG directly reflects brain functional activity. Consequently, EEG-based PD detection has attracted increasing attention. For example, Anjum et al. [25] employed EEG data to detect cognitive impairment in PD patients, and Ly et al. [26] constructed a support vector machine (SVM) model to identify gait initiation failure in PD.

With the development of deep learning, its strong capacity for automatic feature representation and modeling of complex nonlinear patterns has gradually made it the mainstream approach for EEG-based PD detection. Qiu et al. [27] proposed a multiscale convolutional neural network, MCPNet, for PD detection. Similarly, Balaji et al. [28] and Bdaqli et al. [29] developed detection models based on verifiable CNN and CNN-LSTM architectures, respectively. Beyond PD detection, deep learning has been widely applied to EEG signal analysis tasks, including epilepsy detection [30] and depression recognition [31], owing to its effective representation of complex nonlinear signals. Currently, temporal networks suitable for EEG modeling include traditional architectures such as CNN [32], LSTM [33], and Transformer [34], as well as novel structures such as iTransformer [35], ModernTCN [36], and Mamba2 [37]. Moreover, various optimization strategies—including residual connections [38], attention mechanisms [39], and causal dilated convolutions [40]—further enhance the temporal modeling capabilities of these networks.

Despite these advances, two common challenges remain. First, most studies rely on single-domain features extracted either from the time or frequency domain, lacking comprehensive modeling of EEG’s non-stationary and multi-frequency characteristics. For example, Chowdhury et al. [40] utilized only time-domain features, whereas Göker et al. [41] focused exclusively on frequency-domain features. Second, sample sizes are often small, with most studies based on datasets comprising only a few dozen participants. For instance, Salah et al. [42] included only 40 participants (20 PD and 20 HC), and the dataset used by Jibon et al. [11] contained 31 participants (16 HC and 15 PD).

To address the limitations of insufficient multiscale EEG feature modeling and the restricted dataset size, this study constructed a PD EEG dataset comprising 415 participants (289 HC and 126 PD). Furthermore, we propose a three-domain collaborative modeling method, termed MDF-Net, which integrates temporal dynamics, spectral rhythm features, and wavelet-based time–frequency representations. By leveraging cross-domain feature complementarity, MDF-Net effectively enhances feature discriminability and achieves high-precision EEG-based PD detection.

## 3. Dataset and Methods

### 3.1. EEG Dataset and Data Preprocessing

The EEG data utilized in this study were obtained from a PD research project conducted in collaboration with the First Affiliated Hospital of Henan University of Science and Technology. Data collection was carried out from July 2023 to May 2025, including a total of 415 participants, comprising 289 HC and 126 PD patients. The health status and PD diagnosis of all participants were evaluated and confirmed by professional neurologists according to established clinical criteria.

**Figure 1:**
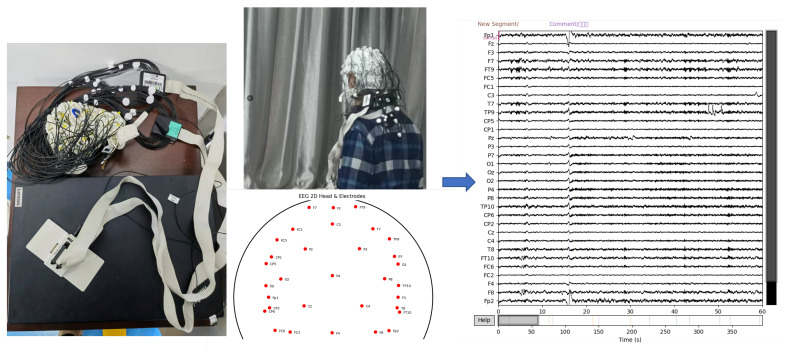
Schematic diagram of the EEG sensor data acquisition process.

The EEG data were collected as part of the study protocol. During data acquisition, participants wore EEG recording equipment and for PD patients, they were instructed not to take medication for 12 h prior to testing, so that PD data were collected in the OFF state. Electrode impedance was checked to ensure proper connectivity. Subsequently, they were instructed to remain relaxed with eyes closed for approximately 5 min while continuous EEG signals were recorded. The EEG recording system employed a 32-channel electrode layout for signal acquisition with a sampling rate of 500 Hz. A schematic illustration of the data acquisition process and the raw EEG signals is shown in Figure 1.

To improve signal quality and remove artifacts, the collected EEG data underwent the following preprocessing pipeline. First, a 1–40 Hz band-pass filter was applied to remove power-line interference and high-frequency noise. Independent component analysis (ICA) was then performed with 15 components per recording to separate artifacts such as ocular and muscular activity. Components whose variance or kurtosis exceeded the 90th percentile were removed, resulting in an average of 3.76 ± 0.45 components removed per recording (range: 2–4). Subsequently, the signals were resampled to a lower sampling rate, amplitude-normalized using Z-score standardization, and segmented to generate samples suitable for model training.

All experimental procedures were approved by the Ethics Committee of the First Affiliated Hospital of Henan University of Science and Technology (Approval No. 2023-03-K0029), and written informed consent was obtained from all participants. The demographic characteristics of the participants are summarized in Table 1.

### 3.2. Network Architecture and Methods

**Figure 2:**
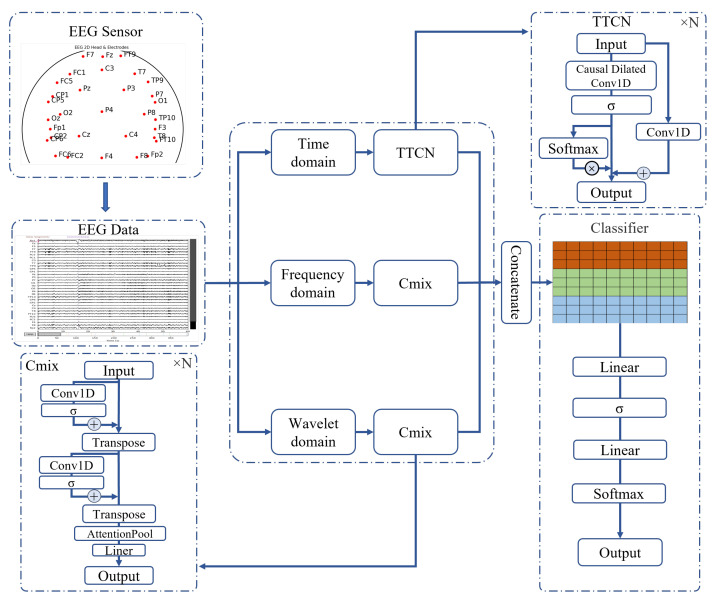
Overall architecture of the Multi-Domain Fusion Network.

In this study, we propose a multi-domain fusion EEG signal classification model, named the Multi-Domain Fusion Network (MDF-Net), which aims to fully exploit the complementary information of EEG signals across the temporal, frequency, and wavelet domains. The MDF-Net adopts a multi-branch architecture, consisting of a Temporal Attention-enhanced Temporal Convolutional Network (TTCN) branch for processing raw time-series data, and two 1D Convolutional Neural Network mixer (Cmix) branches for processing frequency and wavelet features, respectively. This design enables collaborative modeling of multi-domain signal features. The overall architecture of the model is illustrated in Figure 2.

For the input EEG data, three input subspaces are defined according to the transformation domain:

- **Temporal branch:** This branch receives raw time-series EEG data and extracts local temporal features using the TTCN module. The TTCN stacks multiple layers of causal dilated convolutions with exponentially increasing dilation rates, which significantly enlarges the receptive field without increasing the number of parameters, effectively preserving long-range sequential information. Temporal Attention is incorporated to enhance feature representation and improve training stability.
- **Frequency branch:** This branch processes the frequency-domain representation obtained via Fast Fourier Transform (FFT). A lightweight Cmix module is employed to enable feature interaction across time steps and perform channel-wise information fusion.
- **Wavelet branch:** This branch handles features derived from Discrete Wavelet Transform (DWT). Using the same Cmix module as the frequency branch, it further models multi-scale dynamic features to capture local time-frequency variations in the EEG signals.

Within the Cmix module, an attention pooling mechanism is applied to adaptively aggregate features along the temporal dimension. This mechanism allows the model to emphasize key information and enhances the discriminative power of the multi-domain features.

#### 3.2.1. Fourier Transform and Wavelet Transform Modules

**Fourier Transform Modules:** The frequency-domain representation emphasizes the energy distribution across different frequency components, which is particularly useful for characterizing rhythmic neural oscillations in EEG signals and for identifying frequency-specific abnormalities associated with neurological conditions. To obtain the frequency-domain representation of EEG signals, this study applies the FFT to the time-series data. Fourier transform maps a signal from the time domain to the frequency domain, revealing potential periodic oscillatory structures within the signal. Its discrete form, the discrete Fourier transform (DFT), is defined as:

(1) $$X\left(k\right)=\sum_{n=0}^{N-1}x\left(n\right)e^{-j\frac{2\pi }{N}kn},\;k=0,1,\ldots ,N-1$$

where x(n) represents the discrete time-domain signal of length *N*, and X(k) denotes the corresponding complex frequency-domain coefficients.

To improve computational efficiency, FFT is employed to extract frequency-domain features, reducing the computational complexity from $O(N^{2})$ to O(NlogN), making it suitable for processing high-dimensional time-series signals such as EEG. Since frequency-domain analysis typically focuses on the energy distribution of different frequency components, the amplitude spectrum is adopted as the frequency-domain feature, computed as:

(2) |X(k)|=Re(X(k))2+Im(X(k))2

where Re(X(k)) denotes the real part of X(k), corresponding to the cosine component of the signal, and Im(X(k)) denotes the imaginary part, corresponding to the sine component. The resulting amplitude spectrum effectively reflects the strength of neural oscillatory rhythms in EEG signals.

**Wavelet Transform Module:** To further characterize the non-stationary properties of EEG signals at different temporal scales, this study employs DWT to construct time/frequency-domain feature representations. Wavelet transform decomposes a signal using a set of basic functions with scaling and translation properties, preserving both local temporal and frequency information. Compared with both the time-domain representation and the frequency-domain representation obtained via conventional Fourier analysis, the wavelet domain provides unique features that capture the non-stationary and multi-scale characteristics of biological signals such as EEG. These features enable the modeling of transient neural dynamics and cross-frequency activation patterns.

For a discrete EEG signal x(n), a single-level discrete wavelet decomposition can be expressed as:

(3) $$\begin{array}{cc} c_{A}\left(k\right) & =\sum_{n}x\left(n\right)g\left(2k-n\right) \end{array}$$

(4) $$\begin{array}{cc} c_{D}\left(k\right) & =\sum_{n}x\left(n\right)h\left(2k-n\right) \end{array}$$

where g(·) and h(·) are the low-pass and high-pass filters corresponding to the scaling function and wavelet function, respectively. The decomposition splits the original signal into approximation coefficients $c_{A}\left(k\right)$ (low-frequency components representing global trends) and detail coefficients $c_{D}\left(k\right)$ (high-frequency components representing local variations).

In this study, the Daubechies wavelet family, specifically the db4 wavelet, is used as the wavelet basis. Each EEG channel undergoes single-level wavelet decomposition, and the resulting $c_{A}$ and $c_{D}$ coefficients are concatenated to form a time-frequency feature vector:

(5) $$\mathrm{FDWT}=[c_{A}\;||\;c_{D}]$$

This feature preserves the multi-resolution structure of the signal, facilitating the modeling of instantaneous neural variations and cross-frequency activation patterns. It also provides complementary time-frequency information for subsequent feature fusion.

#### 3.2.2. TTCN and Cmix Modules

**TTCN Module:** To extract effective temporal features from EEG signals, this study employs a Temporal Convolutional Network enhanced with Temporal Attention in the TCN temporal branch. The incorporation of Temporal Attention enhances the model’s responsiveness to key temporal segments.

The TCN extracts temporal features via one-dimensional convolutions and employs causal dilated convolutions to expand the receptive field. Let the input signal be $x\in R^{C\times T}$, where *C* is the number of channels and *T* is the temporal length. The TCN with causal dilated convolutions is defined as:

(6) $$y\left(t\right)=\sum_{i=0}^{k-1}x_{t-d_{i}\cdot i}\cdot w_{i}$$

where y(t) is the output at time step *t*, *k* denotes the kernel size, w(i) represents the convolutional kernel weights, *d* is the dilation rate, and $d_{i}=2^{i-1}$ increases exponentially to enlarge the receptive field. The convolution kernel slides only to the left along the temporal sequence.

Each convolutional block output F(x) is added to a residual mapping R(x) and passed through a nonlinear activation σ(·):

(7) $$y_{\mathrm{block}}\left(x\right)=\sigma \left(F\left(x\right)+R\left(x\right)\right)$$

where R(x) is implemented as a 1×1 convolution to adjust the channel dimension and ensure it matches the output.

To emphasize features from critical temporal segments, the TCN output feature map $Y\in R^{C\times T}$ is further processed through the Temporal Attention module:

(8) $$f_{\mathrm{time}}=\sum_{t=1}^{T}\alpha_{t}\cdot Y\left(:,t\right)$$

where the attention weight $\alpha_{t}$ is computed as:

(9) $$\alpha_{t}=\frac{\exp(w^{⊤}\cdot Y\left(:,t\right))}{\sum_{s=1}^{T}\exp\left(w^{⊤}\cdot Y\left(:,s\right)\right)}$$

And *w* denotes a learnable weight vector. The GELU activation function is applied throughout the module. The workflow of the TTCN branch is illustrated in the TTCN component of Figure 2.

**Cmix Module:** To extract informative features from EEG signals in the frequency and wavelet domains, this study designed the Cmix to achieve feature mixing across time steps and channels. This branch is capable of capturing local patterns and cross-channel dependencies in frequency- or wavelet-domain signals.

Let the input feature matrix be $x\in R^{C\times F}$, where *C* is the number of EEG channels and *F* denotes the number of frequency (or wavelet) coefficients. Cmix performs information fusion across time steps and channels using 1D convolutional layers combined with residual connections and nonlinear activations, formulated as:

(10) $$g_{\mathrm{token}}\left(x\right)=x+\sigma \left(x\cdot W_{\mathrm{token}}\right)$$

(11) $$g_{\mathrm{channel}}\left(x\right)=g_{\mathrm{token}}\left(x\right)+\sigma \left(g_{\mathrm{token}}\left(x\right)\cdot W_{\mathrm{channel}}\right)$$

(12) $$F_{\mathrm{mixer}}\left(x\right)=g_{\mathrm{channel}}\left(x\right)$$

where $g_{\mathrm{channel}}\left(x\right)$ aggregates information across frequency or wavelet coefficients, and $g_{\mathrm{token}}\left(x\right)$ aggregates information across channels. The resulting output $F_{\mathrm{mixer}}\left(x\right)$ contains high-order features spanning both temporal and channel dimensions. The activation function σ(·) is implemented using GELU.

Subsequently, attention pooling is applied to obtain the output feature vector:

(13) $$f_{\mathrm{out}}=\sum_{f=1}^{F}\alpha_{f}\cdot F_{\mathrm{mixer}}\left(x\right)\left(:,f\right)$$

(14) $$\alpha_{f}=\frac{\exp(u^{⊤}\cdot F_{\mathrm{mixer}}\left(x\right)\left(:,f\right))}{\sum_{s=1}^{F}\exp\left(u^{⊤}\cdot F_{\mathrm{mixer}}\left(x\right)\left(:,s\right)\right)}$$

where *u* denotes a learnable weight vector.

The overall workflow of the Cmix module is illustrated in the Cmix component of Figure 2.

### 3.3. Evaluation Metrics

To objectively evaluate the model’s performance and generalization ability, 5-fold cross-validation (CV) was employed. Specifically, the dataset was divided into five mutually exclusive subsets of approximately equal size. In each fold, four subsets were used for training, and the remaining subset was used for validation. This procedure was repeated so that each subset served as the validation set once. The training and validation sets were strictly subject-independent, meaning that samples from the same participant were never shared across folds. Unless otherwise specified, all reported performance results represent the average across the five folds.

For the dataset, performing statistically informed splitting is highly meaningful [43]. To ensure balanced data partitions in the 5-CV, subjects were independently split while considering multiple factors. The grouping process prioritized: (1) maintaining a similar number of PD and HC samples per fold, (2) minimizing differences in age distribution, and (3) keeping the gender ratio as consistent as possible. Since the number of EEG segments per subject varied, we performed repeated random splits under these constraints and selected the configuration that achieved the best overall balance. As a result, each fold included approximately 83 subjects, with a male proportion of 0.51 ± 0.05, an overall mean age of 57.30 ± 0.92 years, and mean ages of 55.81 ± 1.10 years for HC and 63.37 ± 1.31 years for PD. These statistics indicate that both age and gender distributions were approximately balanced across folds.

Since each participant may contribute multiple samples, final classification decisions were made at the individual level. A majority voting strategy was adopted, in which the predicted labels of all samples from a participant were aggregated to determine the final decision. In the event of an equal number of votes between classes, the average predicted probability of the samples was used to determine the final classification.

**Figure 3:**
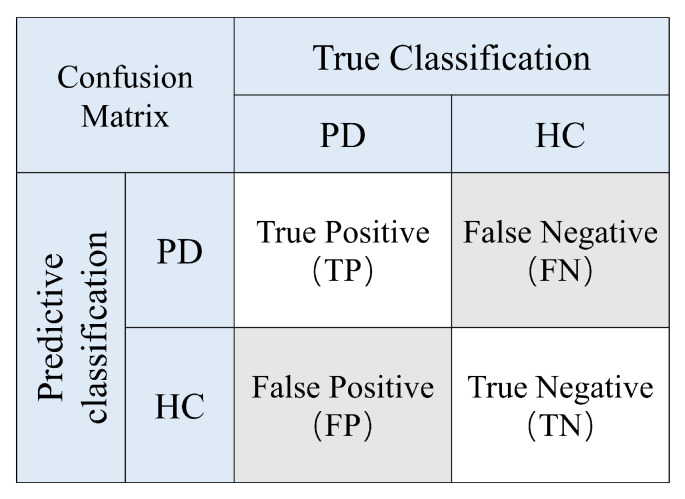
Confusion matrix diagram.

A confusion matrix was constructed for each CV test set, as shown in Figure 3. In the confusion matrix, True Positives (TPs), True Negatives (TNs), False Positives (FPs), and False Negatives (FNs) are reported. The following metrics were derived from the confusion matrix and calculated as follows:

(15) $$\mathrm{Accuracy}\;\left(\mathrm{Acc}\right)=\frac{TP+TN}{TP+TN+FP+FN}$$

(16) $$\mathrm{Precision}\;\left(\mathrm{Prec}\right)=\frac{TP}{TP+FP}$$

(17) $$\mathrm{Recall}\;\left(\mathrm{Rec}\right)=\frac{TP}{TP+FN}$$

(18) $$F1-\mathrm{score}\;\left(F1\right)=2\times \frac{\mathrm{Prec}\times \mathrm{Rec}}{\mathrm{Prec}+\mathrm{Rec}}$$

Additionally, the Area Under the Receiver Operating Characteristic Curve (AUC) was calculated to assess the overall discriminative capability of the model by evaluating the trade-off between the True Positive Rate (TPR) and False Positive Rate (FPR) across all classification thresholds. A higher AUC, approaching 1, indicates superior discriminative performance.

## 4. Experiments and Results

### 4.1. Experimental Setup and Hyperparameters

All experiments were conducted using Python 3.8, and model training and evaluation were implemented based on the PyTorch 2.0.1 deep learning framework. Auxiliary data processing and performance evaluation were performed using scientific computing libraries including Scikit-Learn 1.2.2 and NumPy 1.24.3. The experiments were executed on a workstation equipped with an Intel Core i9-13900K CPU and an NVIDIA RTX 4090 GPU with 32 GB of memory.

During model training, the batch size was set to 512, and the total number of training epochs was 500. The AdamW optimizer [44] was employed, with hyperparameters $\beta_{1}=0.9$ and $\beta_{2}=0.99$. To stabilize optimization and mitigate gradient oscillations during the early training stage, a learning rate warm-up strategy [45] combined with a piecewise decay schedule was adopted. Specifically, the learning rate was linearly increased from $1\times 10^{-9}$ to $1\times 10^{-5}$ during the first 100 epochs, maintained at $1\times 10^{-5}$ for the subsequent 200 epochs, and gradually decayed to $1\times 10^{-7}$ during the final 200 epochs. The model parameters corresponding to the highest validation F1-score were preserved.

To address class imbalance in the dataset, class weighting was applied according to the sample distribution. In addition, label smoothing [46] and L2 regularization were incorporated to alleviate overfitting. All hyperparameters were empirically tuned, and the key configurations used in the experiments are summarized in Table 2.

### 4.2. Performance Evaluation via Five-Fold Cross-Validation

To comprehensively evaluate the generalization capability and robustness of the proposed model, a 5-CV was conducted on the dataset. The classification performance was assessed using Acc, Prec, Rec, F1, and AUC. The detailed results for each fold are summarized in Table 3.

The model demonstrated consistently high performance across all folds, achieving an average accuracy of 92.3%, precision of 88.7%, recall of 86.5%, F1 of 87.3%, and AUC of 0.943. These results indicate that the model not only accurately identifies PD samples but also maintains a balanced precision–recall trade-off, which is particularly valuable given the class imbalance in the dataset, where the ratio of HC to PD samples is approximately 7:3.

**Figure 4:**
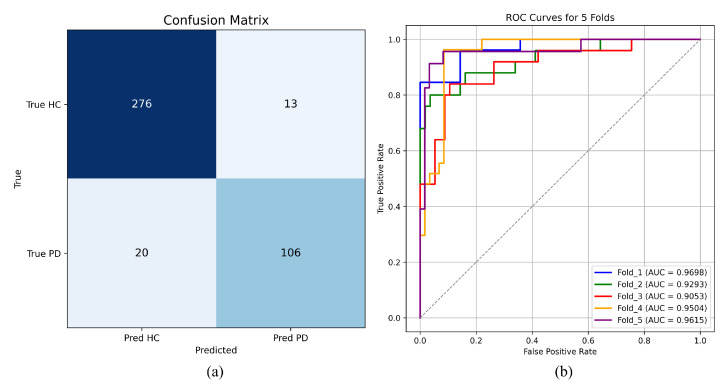
5-CV Confusion matrix and ROC curves. (**a**) Confusion matrix. (**b**) ROC curves.

To further investigate the model’s classification behavior, predictions from all validation folds were aggregated to construct an overall confusion matrix. As illustrated in Figure 4a, the model correctly identified 106 PD samples, with only 20 misclassified as HC. Among the 289 HC samples, only 13 were incorrectly classified as PD, demonstrating that the model achieves reliable discrimination for both classes with a low misclassification rate.

Figure 4b presents the ROC curves obtained from each fold. All curves achieve an AUC greater than 0.90. Although Fold 3 exhibits slightly lower performance compared with the other folds, the remaining four folds reach an accuracy above 90% and an F1-score exceeding 85%. These results confirm the robustness and reliable discriminative capability of the model under the cross-validation setting, indicating strong generalization ability and consistent performance across different data partitions.

### 4.3. Comparison with Multiple Baseline Models

To further validate the effectiveness of the proposed model, its performance was compared with a range of classical and state-of-the-art baseline models, as summarized in Table 4. The comparative models included traditional machine learning methods such as SVM and MLP, classical deep learning architectures including CNN, LSTM, TCN, and Transformer, as well as recently developed models such as iTransform and ModernTCN.

As shown in Table 4, traditional machine learning methods exhibited relatively limited performance, with accuracies of 72.7% and 75.7% for SVM and MLP, respectively, and F1-scores approximately 62%. Classical deep learning models, including CNN, LSTM, and TCN, achieved substantially better results, with accuracies ranging from 86.0% to 87.5% and F1-scores between 77.6% and 80.0%. Compared with the original Transformer, the iTransform model enhanced temporal feature representation, achieving an accuracy of 89.2% and an F1-score of 81.2%. Likewise, the ModernTCN model, an improved variant of TCN, achieved an accuracy of 89.7% and an F1-score of 82.9%.

In comparison, the proposed MDF-Net achieved the best overall performance across all evaluation metrics, with a recall of 86.5%, representing a notable improvement over that of ModernTCN (80.1%). This improvement in recall demonstrates that MDF-Net can more reliably identify PD (positive) samples. The superior performance can be attributed to the integrated utilization of time-domain, frequency-domain, and wavelet-domain features, while the TTCN and Cmix modules further enhance feature representation and effectively mitigate overfitting.

To further compare the effectiveness of MDF-Net and ModernTCN, the performance comparison across 5-CV is summarized in Table 5. MDF-Net achieved higher mean values than ModernTCN on all five evaluation metrics. To assess whether the differences in all metrics (Acc, Prce, Rec, F1, AUC) between the 5-CV results of the two experiments are statistically significant, paired *t*-tests were conducted at a significance level of α=0.05. In this context, the *t*-test evaluates whether the means of two related groups differ significantly, and the *p*-value indicates the probability of observing such a difference by chance. In particular, the improvements in ACC and F1 score were statistically significant (t = 3.31, *p* = 0.0296; t = 3.37, *p* = 0.0280, respectively), indicating that MDF-Net can provide more reliable and balanced classification performance. Although precision and recall of MDF-Net were also higher on average, these differences did not reach statistical significance (*p* > 0.05), possibly due to variability across the 5 folds. The AUC values of both models were nearly identical (0.943 ± 0.03), suggesting that both methods exhibited comparable discriminative ability in distinguishing between classes. Overall, these results demonstrate that MDF-Net offers a significant enhancement in classification accuracy and overall predictive balance compared with the ModernTCN baseline, while maintaining similar discriminative capability.

In summary, these results provide strong evidence of the effectiveness and superiority of the proposed MDF-Net for PD classification using EEG data.

### 4.4. Repeated Independent Validation

**Figure 5:**
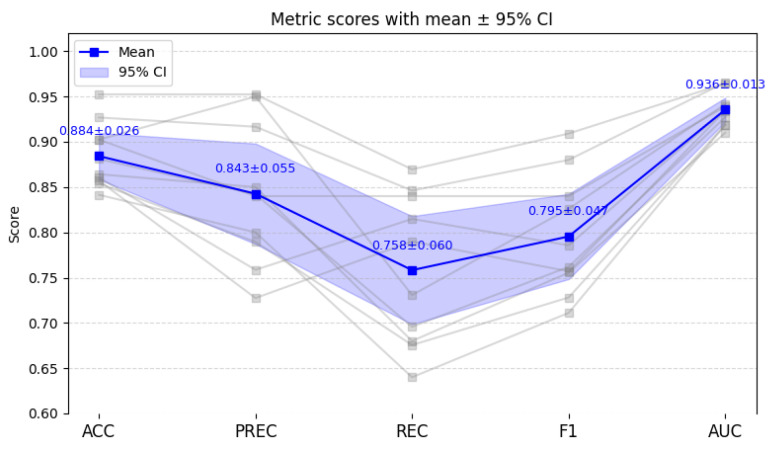
Results of repeated independent hold-out validation.

To further verify the robustness and generalization capability of MDF-Net, we conducted ten independent runs using the hold-out method, in which the dataset was randomly split into 60% training, 20% validation, and 20% testing sets for each run, with no individual appearing in both the training and testing sets within a single run. The corresponding results are illustrated in Figure 5. In the figure, grey lines represent the results of each individual run, the blue line represents the average result, and the blue shaded area indicates the 95% confidence interval.

Based on the repeated hold-out experiments, MDF-Net achieved the following average performance: ACC = 88.4 ± 2.57%, PREC = 84.3 ± 5.55%, REC = 75.8 ± 5.97%, F1 = 79.6 ± 4.70%, and AUC = 0.936 ± 0.013. These findings indicate that MDF-Net maintains strong classification performance and high consistency across multiple random splits, with relatively low variation between runs.

While the mean ACC and F1 scores are slightly lower than those observed in the internal 5-CV, the standard deviations are moderately higher, which can be attributed to the smaller effective training size (reduced from 80% in 5-CV to 60% here) and the independent distribution of the hold-out splits. Nevertheless, the overall performance remains robust, demonstrating that MDF-Net generalizes effectively beyond the original cross-validation folds and is not overly dependent on specific data partitions.

### 4.5. Ablation Study

**Figure 6:**
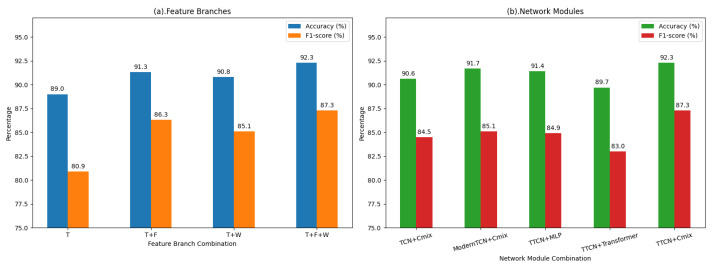
Ablation study of MDF-Net comparison of feature branch combinations (**a**) and network module variants (**b**) on classification performance.

To further investigate the contributions of both feature components and network modules in MDF-Net, we conducted comprehensive ablation experiments using different combinations of time-domain (T), frequency-domain (F), and wavelet-domain (W) features, as well as different network modules for temporal and non-temporal branches. The results are summarized in Table 6 and Table 7, and visually illustrated in Figure 6, which compares Accuracy and F1-score across different feature branch and network module configurations.

When only the time-domain feature was used, the model achieved an accuracy of 89.0% and an F1-score of 80.9%, serving as the baseline performance. Incorporating frequency-domain information (T+F) or wavelet-domain features (T+W) led to notable improvements, suggesting that these auxiliary features provide complementary information that helps capture both global and localized EEG characteristics.

Table 7 compares different modules for temporal and non-temporal branches. TTCN consistently outperforms TCN and ModernTCN in temporal modeling, achieving higher overall metrics. This improvement may be attributed to the relatively short temporal segments of EEG signals, where the incorporation of an appropriately designed attention mechanism allows the model to capture salient temporal dependencies more effectively, outperforming both overly complex models and models without attention. Notably, for non-temporal features, the attention pooling mechanism in Cmix emphasizes the most informative features while suppressing irrelevant or noisy signals, enhancing the quality of frequency and wavelet domain feature fusion. In contrast, Transformer, despite its strong feature representation capacity, can cause partial overfitting with limited data, resulting in lower recall, and MLP underperforms due to insufficient non-temporal feature extraction ability. Overall, the combination of TTCN and Cmix with attention pooling achieves the best balance for multi-domain feature learning, effectively improving discriminative power and generalization.

Overall, the ablation experiments demonstrate that integrating time-domain (T), frequency-domain (F), and wavelet-domain (W) features with TTCN for temporal modeling and Cmix for non-temporal feature fusion achieves the best overall performance, with 92.3% accuracy, 87.3% F1-score, and 0.943 AUC, highlighting the effectiveness of the joint use of three domains and specialized modules in enhancing discriminative power and generalization.

## 5. Discussion

### 5.1. Comparison and Discussion of Studies Using Different Sensors

The experimental results presented above demonstrate that the proposed model achieves outstanding classification performance on EEG data, with an accuracy of 92.3% and an F1-score of 87.3%. To further validate the practical applicability of these findings, and to assess the comparative performance of EEG sensors relative to other sensor modalities in PD detection, we selected several representative sensor studies published in recent years for comparative analysis. The results are summarized in Table 8. It should be noted that, due to differences in datasets, participant cohorts, and experimental settings across studies, the reported accuracy and F1-scores are not directly comparable, though they still provide valuable reference information.

As shown in Table 8, most studies employing wearable motion sensors (e.g., foot-worn devices or inertial measurement units, IMUs) or video-based sensors reported accuracies typically in the range of 80–90%. For example, Oğul et al. obtained an accuracy of 82.0% using foot pressure signals, while He et al. demonstrated 84.1% using video-based gait analysis. Although some IMU-based approaches, such as Bremm et al., reached a relatively high accuracy of 94.2%, their datasets were small (only 45 subjects), and the generalization ability of such models remains uncertain. This is partly because IMU sensors capture limb movement signals, which are relatively low-noise and have simpler patterns, making it easier to achieve high accuracy even with small datasets. In comparison, EEG records brain activity, which is inherently more complex and noisier, with features that are harder to extract. As a result, EEG-based models may sometimes exhibit slightly lower accuracy but offer higher neurophysiological interpretability and provide valuable insights.

In contrast, our EEG-based approach achieved an accuracy of 92.3%, an F1-score of 87.3%, and an AUC of 0.94 on a substantially larger dataset comprising 415 subjects (HC:PD ≈ 7:3), demonstrating greater robustness and model stability. Importantly, this dataset exhibits a higher degree of class imbalance compared with previous studies, better reflecting the real-world clinical distribution, where PD patients are less prevalent than HC.

Although EEG-based detection has certain limitations, such as higher requirements for the recording environment and potential sensitivity to noise, it offers notable advantages in terms of independence from motor tasks. EEG signals directly reflect neural activity associated with central nervous system dysfunction, providing stronger neurophysiological interpretability compared with movement-dependent modalities. Furthermore, unlike gait- or motion-based sensors that require active subject participation, EEG-based detection imposes minimal physical demands, making it particularly suitable for PD patients with severe motor impairments or limited mobility.

Overall, both from the perspectives of performance and practical applicability, EEG-based methods show significant promise for PD detection. The proposed MDF-Net, trained on EEG data, demonstrates strong discriminative capability for PD classification and offers a feasible and noninvasive approach for supporting neurological diagnosis.

### 5.2. Discussion on Sampling Interval and Sampling Frequency

To identify the most effective data input configuration for EEG-based PD classification, we evaluated the effects of varying sampling intervals and sampling frequencies on model performance. The results are summarized in Table 9. In the table, D denotes the sampling interval (in seconds), while S denotes the sampling frequency (the downsampled rate from the original 500 Hz data). Metrics in the sample-level columns represent accuracy calculated per single sample, whereas metrics in the subject-level columns correspond to overall accuracy after majority voting across all samples.

Regarding sampling frequency, when the frequency exceeded 20 Hz, the model performance did not show significant improvement. However, reducing the frequency to 10 Hz resulted in a noticeable decline in performance, indicating that excessively low sampling rates may lead to information loss.

Regarding sampling interval, as the interval decreased from 3 s to 0.5 s, the single-sample accuracy generally increased. In contrast, the overall accuracy after majority voting showed minor fluctuations: it increased from 90.3% at D3 to 92.3% at D1, and slightly decreased to 92.1% at D0.5. Other metrics exhibited similar trends. These results suggest that shortening the sampling interval can enhance the discriminative capability at the subject-level.

We propose that these phenomena may be attributed to the following factors:

- **Enhanced feature stability:** Shorter sampling intervals facilitate the capture of dynamic patterns over consecutive time segments, enabling the model to learn more stable temporal features and thereby improving single-sample recognition performance.
- **Statistical robustness:** The majority voting strategy functions as a simple ensemble approach. By aggregating multiple predictions, it reduces the influence of random misclassifications on the final decision, which can improve the overall accuracy to some extent.

**Figure 7:**
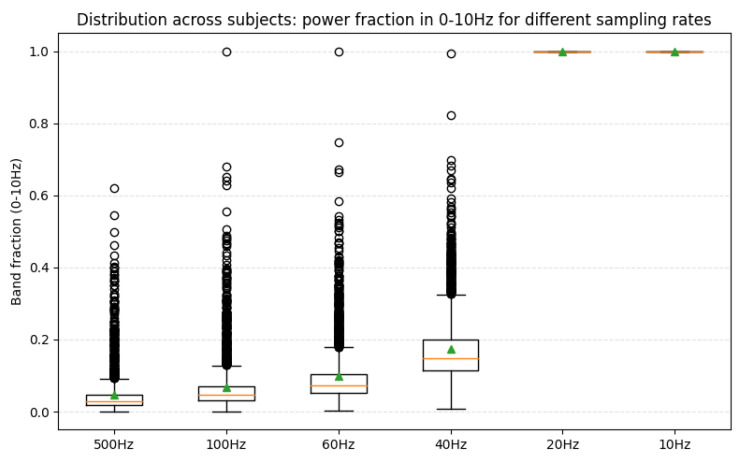
Downsampling of the 0–10 Hz energy. The graph illustrates how the relative energy in the 0–10 Hz frequency band changes under different downsampling rates.

Furthermore, the observation that 20 Hz yielded the optimal performance can be further explained from a frequency-domain perspective [52]. We analyzed the relative power of the 0–10 Hz frequency band under different sampling rates. The average power proportion within this band was only 4.6% at 500 Hz, 6.9% at 100 Hz, 9.8% at 60 Hz, 17.4% at 40 Hz, and 100% at 20 Hz, consistent with the Nyquist sampling theorem [53], as shown in Figure 7.

The importance of the 0–10 Hz range is well supported by previous studies, which have shown that PD-related EEG abnormalities are predominantly concentrated in this frequency region [54]. More recent studies [55,56] also reported that stimulation or modulation below 10 Hz may have beneficial effects for PD patients.

Therefore, our experimental finding that 20 Hz sampling achieved the best performance aligns with both theoretical and physiological evidence. The substantial performance drop observed at 10 Hz suggests that downsampling to this rate discards crucial information between 5–10 Hz, while the lack of improvement at 40 Hz indicates that 20 Hz offers the most effective balance between information preservation and model generalization.

Based on the results, selecting an appropriate sampling interval and sampling frequency is crucial for EEG-based studies, as it can significantly improve model performance. In this study, a sampling interval of 1 s and a sampling frequency of 20 Hz were found to be optimal. According to the Nyquist theorem [53], the theoretical minimum sampling rate for the original EEG sensors is 40 Hz. This insight can inform future work aimed at reducing hardware requirements in the development of EEG-based PD detection devices.

### 5.3. Attention Weight Analysis of MDF-Net

**Figure 8:**
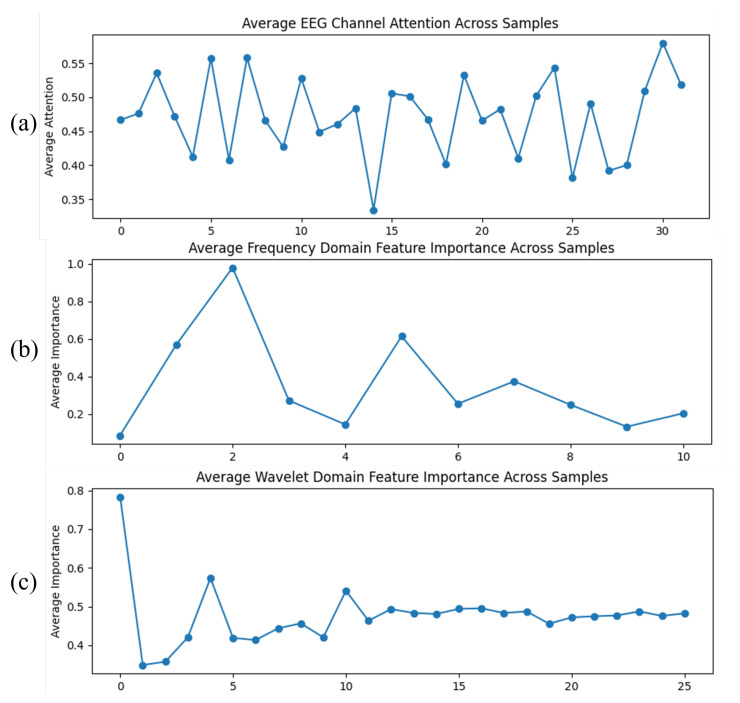
Attention weights of MDF-Net across EEG channels, Fourier frequency bins, and wavelet coefficients. (**a**) EEG channel attention map. (**b**) Fourier domain attention map. (**c**) Wavelet domain attention map.

To gain deeper insight into the discriminative mechanisms of MDF-Net, we visualized the attention weights assigned to 32 EEG channels, 11 Fourier frequency bins, and 26 wavelet coefficients, averaged across all EEG data, as shown in Figure 8.

As shown in the EEG channel attention map (Figure 8a), most channels have weights between 0.4 and 0.55, indicating that the model makes full use of multi-channel information, while being more sensitive to certain specific channels, though the differences are relatively limited.

In the Fourier domain attention map (Figure 8b), the highest-weighted frequency bin is the third bin (with a weight of 0.978), corresponding to a low-frequency component around 2 Hz; the second-highest bin is the sixth bin (with a weight of 0.614), also in the low-frequency range. The remaining bins have weights below 0.4, suggesting that the model primarily relies on low-frequency δ/θ oscillations for classification, with minimal contribution from higher frequencies.

In the wavelet domain attention map (Figure 8c), the highest weight is observed for the first coefficient (0.784), corresponding to the approximate coefficient (lowest frequency), indicating that the model mainly depends on low-frequency temporal information. Other coefficients range between 0.35 and 0.55, showing that the model also leverages some high-frequency or local time-frequency information, but with lower contribution. Overall, the model is highly sensitive to low-frequency temporal features, while high-frequency features serve primarily as auxiliary information.

Taken together, the attention analysis across channels, Fourier, and wavelet domains indicates that MDF-Net mainly relies on low-frequency features and most channels, highlighting its focus on low-frequency EEG oscillations. These results are based on the average attention weights computed across all EEG data, ensuring robustness. This finding is consistent with the conclusions of previous studies [54,55,56,57], indicating that the model primarily attends to low-frequency information. It should be noted that this does not imply that high-frequency features are unaffected by PD; rather, it simply indicates that low-frequency EEG exhibits greater discriminative power when distinguishing HC from PD patients. Due to the characteristics of deep learning models, high-frequency data may be more prone to overfitting, leading to lower assigned weights, although their potential contribution to disease classification cannot be ruled out.

### 5.4. Discussion on Age-Related Effects

To examine the potential influence of age on model performance, an additional subgroup analysis was conducted. Three experiments were designed using different testing age ranges:

1. Both training and testing sets included samples aged 55–60;
2. Training 55–60, testing 60–65;
3. Training 55–60, testing 50–55.

Since the number of available samples varied across age groups, an equal number of samples were randomly selected from each group to ensure balanced training and testing sets. 5-CV was then performed in each case. The specific results are shown in Table 10.

If the model primarily relied on age differences, the performance in experiments 2 and 3 would be expected to decline compared to experiment 1. However, the results remain generally consistent, and even show the highest accuracy when testing on the 60–65 group. This indicates that MDF-Net learns disease-related EEG patterns rather than merely reflecting age differences. The stronger performance in the older group may suggest that PD-related neural abnormalities become more pronounced with age.

Although EEG signals can be influenced by both PD pathology and normal aging, as well as inter-individual variability, disentangling these effects requires a larger and more balanced dataset across age ranges. Future studies will further explore the interaction between aging and PD-related EEG dynamics.

## 6. Conclusions

In this study, we proposed a multi-domain feature fusion model, MDF-Net, for EEG-based signal classification. By integrating complementary features from the time, frequency, and wavelet domains, MDF-Net effectively captures multi-scale dynamic information in EEG signals. The model employs a parallel multi-branch architecture, where the temporal branch incorporates a TTCN to capture correlations across different time scales, while the frequency and wavelet branches Cmix for cross-channel feature interaction and spectral feature fusion. An attention pooling mechanism further enhances the discriminative capability of the extracted features.

Experimental results demonstrate that MDF-Net significantly outperforms models using only time-domain information, achieving an accuracy of 92.3% and an F1-score of 87.3% on our dataset, surpassing most existing deep learning baseline methods. Compared with approaches based on gait or inertial signals, EEG-based detection in this study also exhibits competitive performance, indicating that EEG, as a non-invasive neurophysiological signal, holds considerable potential for PD detection. Furthermore, the relatively large sample size used in this study improves the statistical reliability and generalizability of the results. Overall, the MDF-Net demonstrates strong discriminative capability for PD classification and represents a feasible non-invasive approach for supporting neurological diagnosis.

Future work will focus on three directions. First, we plan to develop a low-cost wearable EEG prototype suitable for clinical PD screening. By reasonably reducing the sampling frequency, hardware complexity and energy consumption can be minimized, making the system more suitable for home use. Moreover, by leveraging cloud or mobile computing for signal analysis and feedback (with participant consent), device costs can be further reduced while continuously improving the model through large-scale data aggregation. Second, we aim to extend the study to multimodal data, incorporating EEG, gait video, speech, and facial expression information to enhance recognition of multidimensional PD symptoms. Third, we will further investigate model interpretability to identify disease-relevant brain activity patterns and potential biomarkers, thereby providing insights into the neural mechanisms underlying PD.

## Figures and Tables

**Table 1:** Demographic Characteristics of Participants (Mean ± SD).

Characteristics	HC	PD
All (Male/Female)	289 (145/144)	126 (71/55)
Age (years)	55.8 ± 9.2	63.8 ± 7.5
Height (cm)	165.5 ± 7.3	165.4 ± 8.5
Weight (kg)	67.5 ± 11.2	66.9 ± 12.0
Hoehn–Yahr stage	N/A	1.9 ± 0.8

**Table 2:** Key hyperparameter settings used in the experiment.

Module	Parameter	Value
EEG preprocessing	Low frequency (Hz)	1
	High frequency (Hz)	40
	Variance percentile	90
	Kurtosis percentile	90
TTCN	Channel dimensions	(64, 128, 64)
	Kernel size	3
	Stride	1
	Dilation rate	$2^{i}$
	Dropout	0.2
Cmix	Channel dimensions	(64, 128, 64)
	Dropout	0.2
Training	Learning rate	1 × $10^{-5}$
	L2 regularization	0.01
	Class weights	$w_{0}=0.8,\;w_{1}=1.2$
	Label smoothing	0.1

**Table 3:** Performance Metrics Obtained from 5-CV.

Fold	Acc (%)	Prec (%)	Rec (%)	F1 (%)	AUC
CV1	95.1	100.0	84.6	91.7	0.970
CV2	91.4	90.9	80.0	85.1	0.929
CV3	87.8	77.8	84.0	80.8	0.905
CV4	91.9	83.3	92.6	87.7	0.950
CV5	95.2	91.3	91.3	91.3	0.961
Mean	92.3	88.7	86.5	87.3	0.943

**Table 4:** Comparison of classification performance among different models.

Model	Acc (%)	Prec (%)	Rec (%)	F1 (%)	AUC
SVM	72.7	74.7	62.9	61.2	0.814
MLP	75.7	78.7	60.7	62.0	0.868
CNN [33]	86.8	80.4	75.2	77.6	0.902
LSTM [32]	86.0	81.5	75.9	78.6	0.898
TCN [16]	87.5	84.4	75.7	80.0	0.903
Transformer [34]	86.7	79.6	76.9	77.6	0.898
iTransform [35]	89.2	87.7	75.9	81.2	0.920
ModernTCN [36]	89.7	86.6	80.1	82.9	0.941
MDF-Net	**92.3**	**88.7**	**86.5**	**87.3**	**0.943**

**Table 5:** Comparison of classification performance between MDF-Net and ModernTCN.

Metric	ModernTCN (Mean ± 95% CI)	MDF-Net (Mean ± 95% CI)	t	*p*
ACC	89.7 ± 4.0%	92.3 ± 3.8%	3.31	0.0296 *
PREC	86.6 ± 6.9%	88.7 ± 10.5%	0.42	0.6954
REC	80.1 ± 11.0%	86.5 ± 6.6%	1.96	0.1213
F1	82.9 ± 6.9%	87.3 ± 5.7%	3.37	0.0280 *
AUC	0.943 ± 0.034	0.943 ± 0.032	0.01	0.9947

* *p* < 0.05, denoting statistical significance.

**Table 6:** Ablation results of MDF-Net under different branch combinations.

T	F	W	Acc (%)	Prec (%)	Rec (%)	F1 (%)	AUC
✓			89.0	82.6	79.2	80.9	0.896
✓	✓		91.3	84.6	**88.9**	86.3	0.937
✓		✓	90.8	85.0	85.8	85.1	0.940
✓	✓	✓	**92.3**	**88.7**	86.5	**87.3**	**0.943**

**Table 7:** Ablation results of different network modules for temporal (T) and non-temporal (F/W) branches.

T	F/W	Acc (%)	Prec (%)	Rec (%)	F1 (%)	AUC
TCN	Cmix	90.6	83.3	85.2	84.5	0.913
ModernTCN	Cmix	91.7	84.7	85.9	85.1	0.939
TTCN	MLP	91.4	84.6	85.1	84.9	0.927
TTCN	Transformer	89.7	87.6	78.6	83.0	0.907
TTCN	Cmix	**92.3**	**88.7**	**86.5**	**87.3**	**0.943**

**Table 8:** Comparison of PD detection performance using different sensors.

Studies	Subjects (HC/PD)	Sensor	Method	Acc (%)	F1 (%)	AUC
Oğul et al. (2021) [47]	73/93	Foot-Worn	SRAnet	82.0	-	0.89
Bremm et al. (2024) [48]	12/33	IMU-Hand	Random Forest	94.2	94.2	-
He et al. (2022) [49]	95/96	Video	ADGCN	84.1	85.8	-
Wang et al. (2022) [50]	30/55	EEG	Capsnet	89.3	-	-
Oh et al. (2020) [51]	20/20	EEG	CNN	88.3	-	-
**Our Study**	289/126	EEG	MDF-Net	92.3	87.3	0.94

If the content is “-”, it indicates that the original paper did not mention this portion of the data.

**Table 9:** Impact of EEG Sampling Interval (D) and Sampling Frequency (S) on Model Performance at Sample- and Subject-Level.

Sampling Setting	Sample-Level					Subject-Level				
	Acc	Prec	Rec	F1	AUC	Acc	Prec	Rec	F1	AUC
D0.5 S20	78.3	59.8	71.1	64.9	0.842	92.1	85.8	88.7	87.1	0.941
D1 S20	79.0	61.5	71.8	66.0	0.846	92.3	88.7	86.5	87.3	0.943
D2 S20	80.2	63.9	69.4	66.5	0.848	91.6	88.0	84.1	85.9	0.934
D3 S20	79.5	61.8	72.5	66.6	0.845	90.3	82.7	86.5	84.5	0.928
D1 S10	76.1	57.2	66.7	61.3	0.814	89.9	84.2	83.5	83.4	0.925
D1 S40	79.0	61.6	69.0	64.9	0.837	91.5	86.2	86.6	86.2	0.930

**Table 10:** Performance comparison across age-subgroup experiments to assess the influence of age on MDF-Net.

Training Age Range	Testing Age Range	Acc	Prec	Rec	F1	AUC
55–60	55–60	87.3%	91.7%	74.3%	80.3%	0.896
55–60	60–65	89.0%	90.5%	81.6%	82.7%	0.891
55–60	50–55	88.1%	78.1%	73.3%	73.7%	0.883

## Data Availability

The data presented in this study are available on request from the corresponding author. The data are not publicly available due to privacy and ethical restrictions.
